# An international invasive meningococcal disease outbreak due to a novel and rapidly expanding serogroup W strain, Scotland and Sweden, July to August 2015

**DOI:** 10.2807/1560-7917.ES.2016.21.45.30395

**Published:** 2016-11-10

**Authors:** Jay Lucidarme, Kevin J Scott, Roisin Ure, Andrew Smith, Diane Lindsay, Bianca Stenmark, Susanne Jacobsson, Hans Fredlund, J Claire Cameron, Alison Smith-Palmer, Jim McMenamin, Steve J Gray, Helen Campbell, Shamez Ladhani, Jamie Findlow, Paula Mölling, Ray Borrow

**Affiliations:** 1 Meningococcal Reference Unit, Public Health England, Manchester, United Kingdom; 2 Scottish Haemophilus, Legionella, Meningococcus and Pneumococcus Reference Laboratory, Glasgow Royal Infirmary, Glasgow, United Kingdom; 3 College of Medical, Veterinary & Life Sciences, Glasgow Dental Hospital & School, University of Glasgow, Glasgow, United Kingdom; 4 National Reference Laboratory for Pathogenic Neisseria, Department of Laboratory Medicine, Faculty of Medicine and Health, Örebro University, Örebro, Sweden; 5 NHS National Services Scotland, Health Protection Scotland, Glasgow, United Kingdom; 6 Immunisation Department, Public Health England, London, United Kingdom

**Keywords:** World, bacterial infections, meningococcal disease, Neisseria meninigitidis, outbreaks, surveillance, epidemiology, molecular methods

## Abstract

The 23rd World Scout Jamboree in 2015 took place in Japan and included over 33,000 scouts from 162 countries. Within nine days of the meeting ending, six cases of laboratory-confirmed invasive serogroup W meningococcal disease occurred among scouts and their close contacts in Scotland and Sweden. The isolates responsible were identical to one-another by routine typing and, where known (4 isolates), belonged to the ST-11 clonal complex (cc11) which is associated with large outbreaks and high case fatality rates. Recent studies have demonstrated the need for high-resolution genomic typing schemes to assign serogroup W cc11 isolates to several distinct strains circulating globally over the past two decades. Here we used such schemes to confirm that the Jamboree-associated cases constituted a genuine outbreak and that this was due to a novel and rapidly expanding strain descended from the strain that has recently expanded in South America and the United Kingdom. We also identify the genetic differences that define the novel strain including four point mutations and three putative recombination events involving the horizontal exchange of 17, six and two genes, respectively. Noteworthy outcomes of these changes were antigenic shifts and the disruption of a transcriptional regulator.

## Introduction

*Neisseria meningitidis* is a leading cause of meningitis and septicaemia [1]. Occurrences of invasive meningococcal disease (IMD) range from sporadic cases to large outbreaks and epidemics. Outbreaks have been associated with mass gatherings such as that of the annual Hajj pilgrimage to Mecca [2]. The 23rd World Scout Jamboree took place between 28 July and 8 August 2015 in Japan and included 33,000 scouts from 162 countries [3]. Over the nine days that followed, three scouts and one non-attending close contact of a healthy scout from the North of Scotland Unit, and two scouts from the Stockholm Unit (Sweden), fell ill with laboratory-confirmed IMD.

All of the patients were admitted to hospital. One of the cases presented with meningitis and shock and was treated in intensive care for six days. The remaining five cases exhibited relatively mild non-specific and/or atypical (respiratory) symptoms. All cases eventually recovered well with no apparent sequelae [3]. A further seven suspected cases among attendees (five in Scotland and two in Sweden, the latter of which represented two further distinct scout units) were eventually discounted. In the course of the outbreak management, chemoprophylaxis was administered to 53 Scottish scouts, leaders and close contacts, and a further individual outside of Scotland. All of them were also offered quadrivalent ACWY conjugate vaccine [4]. In Sweden, where the outbreak initially appeared more diffuse, chemoprophylaxis was offered to all 1,900 Jamboree participants, with an uptake of ca 80% (data not shown). This was accompanied by throat swabbing to assess the meningococcal carriage rate during the outbreak.

Invasive meningococcal isolates (from sterile sites) were obtained from each of the Scottish cases and one of the Swedish cases. The other Swedish case yielded a throat swab isolate and was confirmed as a case serologically by a complement binding assay exhibiting cross-reactivity against *N. meningitidis* and *N. gonorrhoeae*. The six meningococcal isolates were indistinguishable in terms of serogroup and PorA subtype (serogroup W, PorA subtype P1.5,2,36–2). The four Scottish isolates also underwent FetA and multilocus sequence typing (MLST) and, again, were identical to one another (FetA F1–1 and sequence type (ST)-11) [3]. ST-11 is part of the ST-11 clonal complex (cc11) which is associated with multiple serogroups, a tendency to cause outbreaks and epidemics, atypical clinical presentations, and relatively high case fatality rates [5]. Serogroup C cc11, for example, has caused outbreaks among military recruits [6], university undergraduates [7] and more recently, men who have sex with men [8]. Serogroup W cc11 (W:cc11), meanwhile, was responsible for the global Hajj-associated outbreak in 2000 [2], followed by several large epidemics in sub-Saharan Africa recently reviewed by Mustapha et al*.* [9], and the expansion of endemic disease in South Africa [10], South America [11] and Europe [12].

As with the recent scout cases, the vast majority of W:cc11 isolates from each of the above episodes are indistinguishable using routine typing schemes (up to and including the level of MLST) [13]. As a consequence, the organisms responsible have collectively been described as the ‘Hajj strain’, denoting the first large outbreak characterised as such. Relatively high-resolution techniques such as pulsed-field gel electrophoresis, however, were indicative of underlying diversity [14]. More recently, genome-level comparisons have indicated that almost all W:cc11 isolates belong to cc11 lineage 11.1, one of two divergent cc11 lineages. Furthermore, W:cc11 isolates corresponding to the major W:cc11 outbreaks were resolved into distinct clusters (strains) within two divergent lineage 11.1 sublineages [13]. The Hajj strain sublineage comprises the W:cc11 Hajj outbreak strain, sub-Saharan African W:cc11 strains from epidemic periods, and the recent endemic South African W:cc11 strain. The South American W:cc11 strain sublineage (previously designated the ‘South American/United Kingdom (UK) strain’) charts the diversification of the South American strain and closely related UK strain during expansion from southern Brazil, through Argentina and Chile and onto the UK and Europe. Each distinct strain represents clonal expansion from a single ancestor and may be defined by the genetic differences that distinguish it from closely related strains.

The current study sought to determine (i) which of the Jamboree-associated cases represented a genuine outbreak, (ii) to identify the strain/s responsible and its/their relationship to other geo-temporally diverse W:cc11 isolates, (iii) to chart its/their carriage among the Swedish returnees, and (iv) to identify its defining genomic characteristics.

## Methods

### Genomes

The study used all W:cc11 genomes on the PubMLST *Neisseria* database [15] (n = 873; accessed 21/01/16). These included the Scottish (n = 4) and Swedish (n = 2) outbreak isolates and carrier isolates from Swedish Jamboree attendees (n = 10). The latter 16 isolates are hereafter referred to as the ‘Jamboree-associated’ isolates. The W:cc11 panel also included genomes from earlier Scottish (2015, n = 11; 2013, n = 1; and 2012, n = 1) and Swedish (2015, n = 6) cases. A separate subset of sero/genogroup B, C and W cc11 genomes were used as a representative panel spanning the known diversity of cc11 (n = 106; Box) [13].

Box **PubMLST *Neisseria* IDs of a panel of serogroup B, C and W cc11 genomes spanning the known diversity of cc11**

19957, 29677, 29680, 29681, 29683, 21573, 21578, 21582, 21583, 21584, 30087, 30088, 30089, 30090, 30092, 27087, 29679, 29705, 30076, 30077, 19968, 20057, 20154, 20158, 20196, 29633, 29580, 27089, 20066, 29631, 29976, 29664, 21134, 21311, 21330, 26824, 27803, 26733, 26821, 29639, 28103, 21208, 30295, 29571, 30060, 29908, 30284, 29789, 1170, 29590, 29641, 644, 30296, 30244, 29840, 29849, 29858, 29865, 314, 30239, 30240, 30241, 30243, 344, 29611, 29626, 29638, 29891, 21335, 29578, 21196, 29643, 665, 20261, 29831, 30257, 30261, 30260, 21587, 29315, 29329, 29330, 29381, 29324, 29325, 29328, 29331, 29349, 21581, 29334, 29340, 29341, 29366, 29648, 29649, 29651, 29652, 29653, 29709, 29710, 30178, 30234, 30237, 29704, 30183, 30184

Panel selected from [13].

### Genomic analyses

Genome comparisons were performed using the PubMLST genome comparator tool [16]. In order to map their diversity on a ‘macro’ scale, all of the W:cc11 genomes (n = 873) were initially split into two manageable groups and each group, along with the representative panel spanning the known diversity of cc11, underwent genome comparisons in terms of every 50th core gene (numerically) starting with BACT000001. Refined analyses of the population comprising the Jamboree-associated and related genomes were performed using 1,546 core genome loci [12]. Genetic differences defining the Jamboree-associated and related genomes were identified by comparing these and related genomes in terms of all corresponding indexed ‘neis’ loci on the PubMLST *Neisseria* database. Resulting distance matrices were visualised using SplitsTree4 [17].

## Results

In initial comparisons (using 52 core genes) including a panel of isolates representing the known diversity of cc11, the Jamboree-associated isolates were found to cluster with isolates of the South American W:cc11 strain sublineage (data not shown).

**Figure 1:**
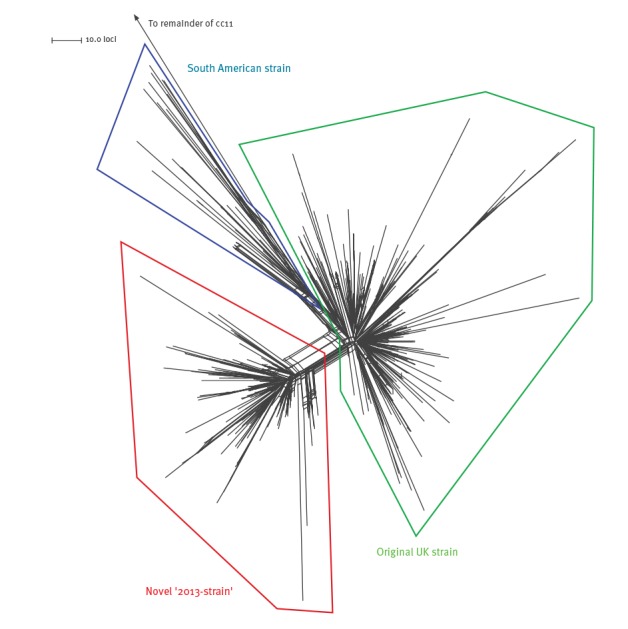
Population structure of the South American W:cc11 strain sublineage

A core genome comparison (1,546 loci) of all of the South American W:cc11 strain sublineage genomes revealed the existence of a novel strain alongside the previously described South American strain and original UK strain that emerged in 2009 [13] (Figure 1).

cc: clonal complex; UK: United Kingdom.

Neighbour-net phylogenetic network based on a comparison of 1,546 core genome loci among all South American W:cc11 strain sublineage genomes (n = 454; accessed on 21 January 2016) on the PubMLST database. A single serogroup B lineage 11.2 genome (M09 240026) was used to represent the remainder of cc11 (‘to remainder of cc11’). The sublineage was divided into three main strains, the South American strain, the original United Kingdom (UK) strain that emerged in the UK in 2009, and the novel ‘2013-strain’ that emerged in the UK in 2013. The scale bar indicates the number of differences among the 1,546 loci compared.

**Figure 2:**
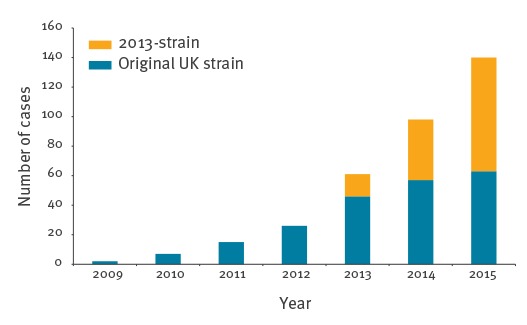
Cases of culture-confirmed invasive meningococcal disease caused by the original United Kingdom strain and 2013-strain of the South American W:cc11 strain sublineage, by year, England, Wales and Northern Ireland, 2009–2015 (n = 349)

The novel ‘2013-strain’ emerged in the UK in 2013 and included all of the Jamboree-associated isolates as well as additional invasive isolates from the UK (2013–2015: 144), France (2015: 3) and Sweden (2015: 6). It also included three UK carrier isolates and a single Finnish isolate (2015) of unknown clinical status. UK cases due to the 2013-strain have approximately doubled year-on-year since its emergence while the initially comparable rate of expansion of the original UK strain began to slow (Figure 2).

cc: clonal complex; UK: United Kingdom.

The chart includes five isolates from pre-Meningococcus Genome Library (2009: 2 and 2010: 3) and includes all corresponding isolates received by the Meningococcal Reference Unit up to 4 November 2015.

**Figure 3:**
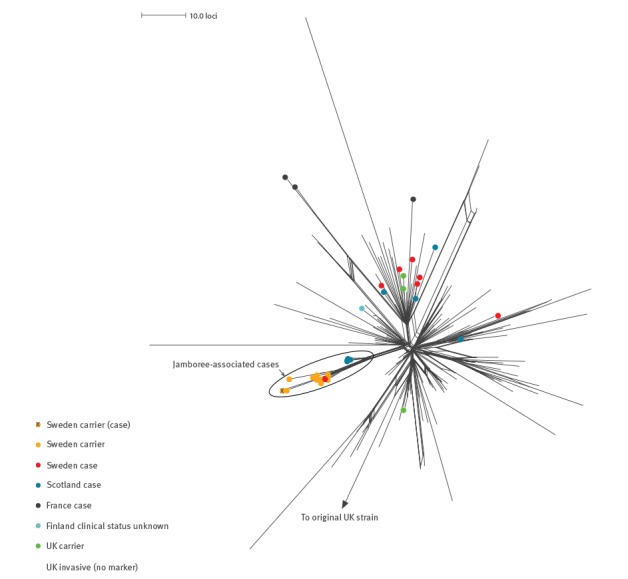
Population structure and geographical distribution of isolates belonging to the 2013-strain of the South American W:cc11 strain sublineage

The 2013-strain isolates and a single original UK strain isolate (M14 240001) underwent a comprehensive genome comparison in terms of all corresponding indexed ‘neis’ loci on the PubMLST *Neisseria* database. The Jamboree-associated isolates exclusively formed a distinct cluster within the 2013-strain (Figure 3).

cc: clonal complex; UK: United Kingdom.

Neighbour-net phylogenetic network based on a comparison of all corresponding indexed ‘neis’ loci among all W:cc11 2013-strain genomes (n = 169) on the PubMLST *Neisseria* database. A single original United Kingdom (UK) strain genome (M14 240001) was used to represent the original UK strain (To original UK strain). The Jamboree-associated isolates (four Scottish cases, two Swedish cases and 10 Swedish carriers) belonged to a distinct cluster – the Jamboree-associated cluster. The other isolates included case isolates from the UK (n = 144; unmarked), France (n = 3) and Sweden (n = 6), carrier isolates from the UK (n = 3), and a Finnish isolate of unknown status. The scale bar indicates the number of differences among all corresponding indexed ‘neis’ loci.

Within the ‘Jamboree-associated cluster’ the Scottish case isolates and Swedish case/carrier isolates formed separate subclusters relatively close/distant to the origin of the main cluster, respectively.

All isolates belonging to the original UK and 2013-strains were compared in terms of all corresponding indexed ‘neis’ loci, as above. The genome comparator output data were examined for common differences distinguishing the 2013-strain from the original UK strain. The transition included three putative recombination events involving 17, six and two genes, respectively, and four point mutations (Table).

**Table:** Common genetic differences distinguishing the 2013-strain from the original UK strain (grouped into putative recombinations where appropriate)

Gene^a^	MC58 identifier^b^	Gene product	Impact^c^
NEIS0813	NMB0872	Putative periplasmic protein	None
NEIS0814	NMB0873	Outer membrane lipoprotein LolB	1 aa change
NEIS0815	NMB0874	4-diphosphocytidyl-2-C-methyl-D-erythritol kinase	16 aa changes
NEIS0816	NMB0875	Ribose-phosphate pyrophosphokinase	None
NEIS0817	NMB0876	50S ribosomal protein L25 (*rplY*)	None
NEIS0818	NMB0877	Putative D-alanyl-D-alanine carboxypeptidase	5 aa changes
NEIS0819	NMB0878	Threonine dehydratase	2 aa changes
NEIS0820	NMB0879	Putative sulphate permease ATP-binding protein	1 aa change
NEIS0821	NMB0880	Putative sulphate permease inner membrane protein	2 aa changes
NEIS0822	NMB0881	Sulphate permease inner membrane protein (*cysU*)	1 aa change
NEIS0823	NMB0882	Hypothetical protein	1 aa change
NEIS0824	NMB0883	Hypothetical protein	2 aa changes
NEIS0825	NMB0884	Superoxide dismutase (*sodB*)	2 aa changes
NEIS0826	NMB0885	Replicative DNA helicase	2 aa changes
NEIS0827	NMB0886	Type IV biogenesis protein (*pilH*)	5 aa changes
NEIS0828	NMB0887	Type IV biogenesis protein (*pilI*)	1 aa change
NEIS0829	NMB0888	Type IV biogenesis protein (*pilJ*)	2 aa changes
NEIS1131	NMB1231	Putative ATP-dependent protease	1 aa change
NEIS1132	NMB1232	Hypothetical protein	In-frame gene acquired^d^
NEIS1133	NMB1233	Exodeoxyribonuclease V alpha subunit	11 aa changes
NEIS1134	NMB1234	Putative ABC-transporter ATP-binding protein	7 aa changes
NEIS1135	NMB1235	Putative integral membrane protein	4 aa changes
NEIS1136	NMB1236	Hypothetical protein	None
NEIS1351	NMB1418	Lipid A biosynthesis lauroyl acyltransferase (lpxL)	1 aa change^e^
NEIS1386	NMB1448	DNA polymerase IV	1 aa change^e^
NEIS1412	NMB1475	Hypothetical protein	1 aa change^e^
NEIS1635	NMB1717	Transcriptional regulator (*mtrR*)	Frameshift^f^
NEIS1946	NA	Haemoglobin-haptoglobin utilisation protein (*hpuA*)	25 aa changes^g^
NEIS1947	NA	Haemoglobin-haptoglobin utilisation protein (*hpuB*)	44 aa changes
NEIS2162	NA	Glycosyltransferase (*csw*)	2 aa changes^h^

NA: not applicable; UK: United Kingdom.

^a^ PubMLST *Neisseria* database identifier.

^b^ MC58 strain identifier, GenBank accession number AE002098.2.

^c^ Regarding predominant alleles for respective strains.

^d^ Gene frameshifted in original UK strain.

^e^ Single nt polymorphism.

^f^ Single bp insertion.

^g^ If homopolymer normalised and within frame.

^h^ Two existing alleles; two compensatory frameshifts.

Genes affected included those encoding antigens (including the haemoglobin-haptoglobin receptor complex HpuAB), the genetic regulator MtrR, and a number of housekeeping/metabolic genes. In addition, the predominant *csw* (serogroup W determinant) genes of the respective strains differed by two compensatory frameshift mutations. As one of these was in a homopolymer, it is uncertain whether these constituted spontaneous mutations or a small recombination event.

Allelic variants within the largest recombinant region (nmb0813 to 0829) ranged from being unique to the W:cc11 isolates (neis0813, neis0815, neis0819, neis0825 and neis0827 to 8) to being observed among isolates belonging to various ccs on the PubMLST database. BLAST searches on the NCBI nt database failed to identify exact matches for the unique W:cc11 alleles. Four out of six alleles within the second largest recombinant region (neis1131 to neis1136) were also observed among multiple ccs. Of the remaining two, neis1131 was unique to the W:cc11 isolates. Only three non-cc11 isolates within the database matched all five of the non-unique alleles – isolate IDs 40007 and 40393 (both ST-10144; 1 invasive and 1 not specified) and isolate ID 20026 (ST-9880; invasive). The acquired *hpuA* and *hpuB* alleles were novel on both the PubMLST and nt databases.

## Discussion

High resolution genomic analyses indicated that the Scottish and Swedish IMD cases associated with the 23rd World Scout Jamboree constituted a genuine outbreak with transmission of meningococci belonging to a distinct phylogenetic cluster over a short period of time.

Isolates from Scottish cases were relatively closely related to one-another, probably reflecting prolonged intragroup contact. Isolates from the Swedish cases and carriers were similarly grouped but at a more distal location within the overall cluster, probably reflecting the spread of carriage among the wider Jamboree participants and further group-wise propagation. Broader dissemination of organisms belonging to the cluster was evident from the Scottish case that occurred in a non-attending close contact. None of the 62 W:cc11 submissions made to the PubMLST *Neisseria* database subsequent to the outbreak, including post-Jamboree cases from Sweden (2015: 2 and 2016: 7), the UK (2015: 33), and France (2016: 11), have, however, belonged to the Jamboree-associated cluster (accessed 20 April 2016; data not shown). Ongoing and retrospective genomic surveillance will determine whether the public health interventions employed in the respective countries have served to curtail onward transmission of organisms belonging to the outbreak cluster.

The Jamboree-associated cluster formed part of a novel strain, the proposed ‘2013-strain’, which emerged in the UK in 2013, with cases approximately doubling annually. This strain represented clonal expansion from a single descendant, or close relative, of the original UK strain which emerged in England in 2009 exhibiting (initially) a similar rate of expansion. The expansion of serogroup W disease in Scotland became evident from 2014 [18], however, the present study identified earlier cases caused by the original UK strain (2012: 1) and 2013-strain (2013: 1), respectively. Prior to the case in 2012, Scotland experienced no W:cc11 cases for at least three years. After 2013, endemic Scottish W:cc11 cases were distributed among both strains. Sweden experienced a greater than three-fold rise in W:cc11 cases in 2015 mainly due to the 2013-strain (n = 7), with a single additional isolate from the Hajj strain sublineage. The 2013-strain was also responsible for the only two Swedish W:cc11 cases in 2014 (data not shown). Prior to 2014, Sweden experienced one confirmed W:cc11 case per year dating back to 2010 (corresponding strains unknown).

Despite cc11 having been associated with numerous focal outbreaks in the past [2,6,7], to our knowledge the original UK strain has only been associated with a single focal outbreak, namely two cases in a healthcare setting in the UK [12,19], with a further four suspected UK outbreaks discounted using the methods described herein (data not shown). The rapid expansion of 2013-strain cases and a possible association with outbreaks may represent heightened carriage, transmission, invasiveness or virulence of the novel strain, or indeed a combination of these factors. This change in epidemiology may, in turn, be a direct consequence of the genetic changes that define the strain. Studies of previous outbreaks/expansions have implicated antigenic shifts in prominent antigens such as PorA [20,21] or fHbp [22], owing to recombination events. As such, possible candidates among 29 altered genes within the 2013-strain include *hpuA* and *hpuB* (encoding the haemoglobin-haptoglobin receptor, HpuAB [23]) which underwent the greatest number of amino acid changes (25 and 44, respectively). Genes involved in the surface expression of other proteins may also be implicated such as the three genes involved in type IV pilus biogenesis (neis0827–8) [24]. Indeed, haemoglobin receptors and pili are not only major antigens but also important virulence factors. Interestingly, relatively remote genes involved in the translocation of lipoproteins to the outer membrane (neis0814, neis0815 and neis1134) were affected by two of the three putative recombination events.

The acquisition of a frameshifted *mtrR* allele may be significant. MtrR is a transcriptional regulator concerned with the expression of various genes in *N. gonorrhoeae*, including those encoding multidrug efflux pumps and others involved in stress responses [25]. It has also been proposed that mutant (including frameshifted) *mtrR* alleles may be advantageous for *N. gonorrhoeae* during infection [25]. MtrR has also recently been implicated in the regulation of *nadA* expression in the meningococcus. NadA is a major surface antigen and a virulence factor involved in adherence and invasion [26,27]. It is also a component of the multicomponent vaccine developed to target serogroup B meningococci (Bexsero) and the likely target of corresponding protection that has been demonstrated against isolates of the original UK W:cc11 strain [28].

The involvement of the *lpxL* gene is noteworthy because this gene is involved in acylation of endotoxin. Frameshifts in *lpxL* have, for example, been implicated in milder disease [29] which was a feature among the Scottish cases but not the Swedish cases, one of whom required six days of intensive care [3]. In the course of routine serogrouping, no obvious effect was observed for the altered *csw* gene. The Public Health England serogrouping assay [30] would not, however, be expected to identify subtle qualitative/quantitative differences in capsule composition.

We were unable to identify potential donor strains involved in several of the putative recombinations and those that we did identify did not belong to common invasive lineages among countries regularly submitting genomic data to the NCBI nt or PubMLST *Neisseria* databases. Other *Neisseria* species less well represented on the sequence databases also constitute potential donors [31]. The acquisition of relatively rare alleles, especially those relating to surface antigens may be advantageous owing to the naivety of the human host population. Genomic analyses of recent carriage studies may shed further light on the identity of the respective donor strains [32,33].

The exact cause of the expansion of the 2013-strain may never be known. Indeed, it may be that this strain has by chance encountered several environments conducive to widespread transmission, such as universities and mass gatherings. Nonetheless, the current analyses revealed that that the continued expansion of W:cc11 in the UK is largely due to the 2013-strain while the expansion of the original UK strain appears to have slowed. Whether the 2013-strain is destined to follow a similar course may also not be known since it is hoped that the recent introduction of the quadrivalent ACWY conjugate vaccine to UK adolescents, including new university entrants, will lead to wider herd protection [34]. Within the 2013-strain, the appearance of the Jamboree-associated cluster appears to have been transient. Should it re-emerge to expand in a way that is comparable to either the 2013- or original UK strains then further investigation may be warranted to identify its defining genetic changes.

The present study demonstrates the utilisation of genomic analysis, in conjunction with comprehensive geo-temporally diverse genomes, to identify bacterial outbreak strains within highly clonal populations. It also demonstrates how such studies may shed light on the emergence of outbreak strains, inform immunisation policy, and, perhaps, inform the development of new vaccines and even therapeutics.
